# Predicting microvascular invasion in hepatocellular carcinoma: a deep learning model validated across hospitals

**DOI:** 10.1186/s40644-021-00425-3

**Published:** 2021-10-09

**Authors:** Shu-Cheng Liu, Jesyin Lai, Jhao-Yu Huang, Chia-Fong Cho, Pei Hua Lee, Min-Hsuan Lu, Chun-Chieh Yeh, Jiaxin Yu, Wei-Ching Lin

**Affiliations:** 1 AI Innovation Center, China Medical University Hospital, Taichung, Taiwan; 2 Department of Medical Imaging, China Medical University Hospital, Taichung, Taiwan; 3 Department of Surgery, Organ Transplantation Center, China Medical University Hospital, Taichung, Taiwan; 4 Department of Medicine, School of Medicine, China Medical University, Taichung, Taiwan; 5 Department of Surgery, Asia University Hospital, Taichung, Taiwan 41354; 6 Department of Biomedical Imaging and Radiological Science, School of Medicine, China Medical University, Taichung, Taiwan

**Keywords:** Hepatocellular carcinoma, Microvascular invasion, Deep learning, External validation

## Abstract

**Background:**

The accuracy of estimating microvascular invasion (MVI) preoperatively in hepatocellular carcinoma (HCC) by clinical observers is low. Most recent studies constructed MVI predictive models utilizing radiological and/or radiomics features extracted from computed tomography (CT) images. These methods, however, rely heavily on human experiences and require manual tumor contouring. We developed a deep learning-based framework for preoperative MVI prediction by using CT images of arterial phase (AP) with simple tumor labeling and without the need of manual feature extraction. The model was further validated on CT images that were originally scanned at multiple different hospitals.

**Methods:**

CT images of AP were acquired for 309 patients from China Medical University Hospital (CMUH). Images of 164 patients, who took their CT scanning at 54 different hospitals but were referred to CMUH, were also collected. Deep learning (ResNet-18) and machine learning (support vector machine) models were constructed with AP images and/or patients’ clinical factors (CFs), and their performance was compared systematically. All models were independently evaluated on two patient cohorts: validation set (within CMUH) and external set (other hospitals). Subsequently, explainability of the best model was visualized using gradient-weighted class activation map (Grad-CAM).

**Results:**

The ResNet-18 model built with AP images and patients’ clinical factors was superior than other models achieving a highest AUC of 0.845. When evaluating on the external set, the model produced an AUC of 0.777, approaching its performance on the validation set. Model interpretation with Grad-CAM revealed that MVI relevant imaging features on CT images were captured and learned by the ResNet-18 model.

**Conclusions:**

This framework provide evidence showing the generalizability and robustness of ResNet-18 in predicting MVI using CT images of AP scanned at multiple different hospitals. Attention heatmaps obtained from model explainability further confirmed that ResNet-18 focused on imaging features on CT overlapping with the conditions used by radiologists to estimate MVI clinically.

## Introduction

Hepatocellular carcinoma (HCC) is a common cancer existing globally and it is now ranked as the fourth major cause for cancer-related death [1, 2]. Currently, liver transplantation (LT), surgical resection and radio-frequency ablation (RFA) are the three potentially curative therapies for HCC [3]. Despite undergoing these treatments, the 5-year recurrence rates after LT, surgical resection and RFA are 10-15 %, 42-52 % and 42-70 %, respectively [1, 4, 5]. It was reported that HCC with microvascular invasion (MVI) positive often recurs within 2 years [3]. It was reported by several studies conducted research on MVI that MVI provides an independent risk factor for predicting tumor recurrence and overall survival rate after resection [6–8]. However, the accuracy of estimating MVI preoperatively by clinical observers is usually low [9].

Previous studies demonstrated preoperative prediction of MVI using computed tomography (CT) images and clinical factors (CFs) [10–12]. Ma et al. (2019) used least absolute shrinkage and selection operator (LASSO) method for radiomic feature extraction and multivariable logistic regression analysis for CF predictor selection to construct models for preoperative MVI prediction. Xu et al. (2019) applied a feature selection support vector machine (SVM) on CT radiomics and then combined with radiological features and CFs (*e.g.* age, gender, Child-Pugh class, hepatic virus infection, etc.) to develop a computational approach for prediction of MVI status and long-term clinical survival outcome of patients with HCC. Peng et al. (2018) developed a predictive model using multivariable logistic regression for MVI status in hepatitis B virus-related HCC patients by including radiomics and CFs. In addition to radiomic and radiological features, Banerjee et al. (2015) [1] demonstrated accurate prediction of histological MVI using radiogenomic venous invasion (RVI) as a contrast-enhanced CT biomarker of MVI. RVI was derived from an association mapping of CT imaging traits with the expression of 91 HCC-specific “venous invasion” gene signatures. They reported that RVI is associated with early disease recurrence and lower overall survival.

Feature-based method is one of main approaches to construct MVI predictive models [10, 12–14]. For instance, potential radiomic features extracted from CT images were selected using supervised or unsupervised methods before the development of predictive statistical or machine learning models. However, shortcomings are identified in these methods. First, the types of features extracted with handcrafted method and the numbers of selected radiomic features included in model development varied from study to study. Using hand-crafted method to extract features from CT images is usually tedious and relies heavily on the experiences of observers. Some underlying imaging information relevant to HCC may not be faithfully captured by observers who are less experienced. Second, these methods were not able to take into account the detailed information provided in the images pixel by pixel. Third, some studies [10, 12, 13, 15] used images with tumor contouring performed manually by radiologists. This, however, will increase workload of radiologists and consume a lot of time in tumor contouring when a predictive model developed with such images was added into the clinical workflow. Manual tumor contouring may also generate selection bias and influence the performance of predictive models. In addition, external validation of predictive model performance using CT images obtained from multiple different medical centers was limited. This restricts the deployment of a well-developed model at other hospitals that use different scanning parameters than the medical center that built the model.

To tackle the above limitations, a deep learning-based framework, in which feature extraction is performed automatically, is demonstrated in this study. A pretrained convolutional neural networks (CNNs) model was applied to analyze HCC histopathological CT imaging features and concatenate with CFs to predict MVI preoperatively. In addition to using CT images from one hospital for model training and validation, external validation of predictive models using CT images obtained across hospitals were also performed. This provided evidence verifying the robustness of our constructed CNN model in generalization and its capability to be applied on CT images obtained at other hospitals. Furthermore, model interpretability was revealed by adopting activation heatmaps to visualize and verify whether the section focused by the model for MVI prediction was in line with the clinical decision workflow performed by radiologists during MVI diagnosis. As a whole, this study provides a new milestone for future deep learning-related research to predict MVI in early-stage HCC across hospitals.

## Materials and methods

### Patients

CT images of HCC patients were collected retrospectively from China Medical University Hospital (CMUH). All HCC cases occurred within the period of Jan, 2007 to Dec, 2020 were identified from the electronic medical record of CMUH. The following inclusion criteria were used: (1) surgical resection or LT for initial MVI diagnosis was performed; (2) HCC was confirmed and MVI status was recorded in the pathological report; (3) clinical data, including age, gender, maximum tumor diameter (MTD), Child-Pugh score, alpha-fetoprotein (AFP), hepatitis B and C status, were available one week before surgery; (4) dynamic CT images included pre-contrast enhancement, late arterial phase (AP) and portal venous phase (PVP) acquired within 3 months before surgery; and (5) presence of a single tumor and no gross venous invasion. Subsequently, HCC cases were excluded with the following criteria: (1) conducted locoregional therapy (*i.e.*, ablation, trans-arterial chemoembolization or radiation therapy) before the time of imaging; (2) presence of other malignant liver tumors; and (3) presence of two or more HCC tumors. Finally, a total of 309 HCC patients (232 men and 77 women) were found to fulfill these exclusion and inclusion criteria. While these patients had their initial CT scanning and subsequent follow-ups at CMUH, another cohort of patients who had their initial CT scanning at other hospitals but were referred to CMUH were also screened with these exclusion and inclusion criteria. A total of 164 HCC patients (127 men and 37 women) were obtained eventually. Since these patients were referred from 54 hospitals and took their first CT imaging outside of CMUH, they were eligible to be used for external validation of model performance.

Before model development, the 309 patients were randomly split at a ratio of 70: 30 into training (*N* = 216) and validation (*N* = 93) sets. Since one patient might have multiple CT slices, we separated our data set based on patients to avoid distributing CT slices of the same patient into both the training and validation sets (*i.e.*, data leakage). Random separation on patient data at a ratio of 70:30 was repeated many times to generate multiple combinations of training and validation sets, each having different numbers of CT slices in both sets. The combination with more CT slices in the training set, and no significant differences of MVI status and CFs in both sets was selected for model development. The demographics of CFs for the training and validation sets are shown in Table 1. On the other hand, the 164 patients, who were referred from other hospitals, were used as the external validation set and their demographics of CFs are shown in Table 2.

**Table 1:** The demographics of clinical factors for patients in the training and validation sets

	Training Set (*N* = 216)		Validation Set (*N* = 93)		*p*-value
	MVI+ (*N* = 68)	MVI- (*N* = 148)	MVI+ (*N* = 28)	MVI- (*N* = 65)	0.41
Median of age in years (Q.25-Q.75)	58 (53–68)	60 (50–68.3)	65 (58.3–72.5)	59 (54–67)	0.32
Gender					0.12
Male	55 (80.9%)	103 (69.6%)	21 (75%)	53 (81.5%)	
Female	13 (19.1%)	45 (30.4%)	7 (25%)	12 (18.5%)	
Median of MTD in mm (Q.25-Q.75)	4.5 (3–7.4)	2.2 (1.5–3.4)	5.9 (3.9–6.9)	2.5 (2–4.2)	0.12
Median of AFP in ng/ml (Q.25-Q.75)	33.5 (6.5–1294.9)	11.4 (3.7–209.6)	9.2 (3.8–328.1)	7 (3.13–71.85)	0.87
Child-Pugh score					0.12
A	64 (94.1%)	139 (93.9%)	25 (89.3%)	59 (90.6%)	
B	4 (5.8%)	7 (4.7%)	2 (7.1%)	4 (6.2%)	
C	0 (0%)	2 (1.4%)	1 (3.6%)	2 (3.1%)	
HBsAg					0.22
Positive	37 (54.4%)	78 (52.7%)	11 (39.3%)	43 (66.2%)	
Negative	31 (45.6%)	70 (47.3%)	17 (60.7%)	22 (33.8%)	
HCsAg					0.27
Positive	23 (33.8%)	52 (35.1%)	11 (39.3%)	18 (27.7%)	
Negative	45 (66.2%)	96 (64.9%)	17 (60.7%)	47 (72.3%)	

Data are presented as n (%) unless otherwise noted. *P*-value were derived from statistical comparison between the training and validation sets using Mann–Whitney–Wilcoxon text,

*p*-value *>* 0.05 indicates no significant difference exists between these two data sets. *MVI+* microvascular invasion positive; *MVI*- microvascular invasion negative; *MTD* maximum tumor diameter; *AFP* alpha-fetoprotein; *Q.25* 25 % quantile; *Q.75* 75 % quantile; *HBsAg* Hepatitis B surface antigen; *HCsAg* Hepatitis C surface antigen

**Table 2:** The demographics of clinical factors for patients who were included in the external set

	External validation set (*N* = 164)	
	MVI+ (*N* = 39)	MVI- (*N* = 125)
Median of age in years (Q.25-Q.75)	62 (54.5–69.5)	63 (57–71)
Gender		
Male	32 (82.1%)	95 (76%)
Female	7 (17.9%)	30 (24%)
Median of MTD in mm (Q.25-Q.75)	5.8 (4.25–8.15)	3.1 (2.3–5.2)
Median of AFP in ng/ml (Q.25-Q.75)	28.7 (3.13–339.83)	6.63 (2.94–62.47)
Child-Pugh score		
A	32 (82.1%)	115 (92%)
B	6 (15.4%)	7 (5.6%)
C	1 (2.6%)	3 (2.4%)
HBsAg		
Positive	14 (35.9%)	59 (47.2%)
Negative	25 (64.1%)	66 (52.8%)
HCsAg		
Positive	16 (41%)	43 (34.4%)
Negative	23 (59%)	82 (65.6%)

Abbreviations are similar to Table 1.

### Feature selection and statistical analysis for patients’ clinical factors

According to some previous studies [10, 12, 16], age, gender, maximum tumor diameter (MTD), Child-Pugh score, AFP, and hepatitis B status were the common features used to estimate MVI. In additional to hepatitis B, some patients enrolled in this study were found to have hepatitis C as well. As a consequence, we decided to include gender, age, MTD, AFP, Child-Pugh score, hepatitis B and C status as patients’ CF for model development. Subsequently, a multivariate logistic regression analysis was performed on the association of these CFs with MVI status.

### CT images

All CT images were scanned using multi-detector CT scanners (Brightspeed 16:

N=91, Lightspeed 16: N=45, Lightspeed VCT 64: N=46, and Optima 660: N=126, GE Medical Systems, Milwaukee, WI) except one case, which was scanned with 640-row spiral CT scannerk (Aquilion ONE, Canon medical systems, Hong Kong). A 1.5 mL/kg body weight bolus of Iohexol or Iodixanol (Omnipaque or Visipaque, GE Healthcare.) was injected intravenously via a power injector at a flow rate of at least 2.7 mL/s. After pre-contrast enhanced CT images were captured, a smart prepared technique was used. This technique involves repeatedly scanning the aorta after contrast medium injection and waiting for the density of aorta to rise over 120 Hounsfield unit (HU) before started scanning the AP images. During scanning, a target region was placed at the abdominal aorta level located above the orifice of celiac artery. PVP images were obtained at 8-15 seconds after AP, and delay phase (DP) images were obtained at 80-90 seconds after PVP. The scanning parameters were 120 kV, auto current in mA, 0.8 s rotation time and a collimation of 1.25 mm. Axial slices were reconstructed with a slice width of 5.0 mm and a slice interval of 5.0 mm. CT scanning protocols, which were performed at other hospitals, for patients in the external validation set contained pre-contrast enhanced CT, AP and PVP. However, the scanning parameters used by other hospitals might vary from one another.

Since the process of scanning AP images, which started after the density of aorta rise over 120 HU, was consistently performed in all hospitals and the timings of scanning PVP and DP images are often different across hospitals, we decided to use CT images of AP (not DP or/and PVP) to eliminate data drift in CT images due to inconsistent imaging parameters applied by distinct medical centers. A total of 1927 CT slices of AP were collected from the 309 patients in the training and validation sets, in which 216 patients in the training set contained 1186 slices (MVI+: 578; MVI-:608) and 93 patients in the validation set contained 741 slices (MVI+: 377; MVI-: 364). For the external validation set, a total of 1418 CT slices (MVI+:505; MVI-: 913) of AP were obtained from the 164 patients. All slices were confirmed to contain only one HCC tumor. All slices had a resolution of 512 × 512 with grayscale values ranging from 0 to 255. The grayscale values were transformed from HU by applying a linear transformation with a window level of 70 and a window width of 200.

### Labeling of regions of interest

**Fig. 1:**
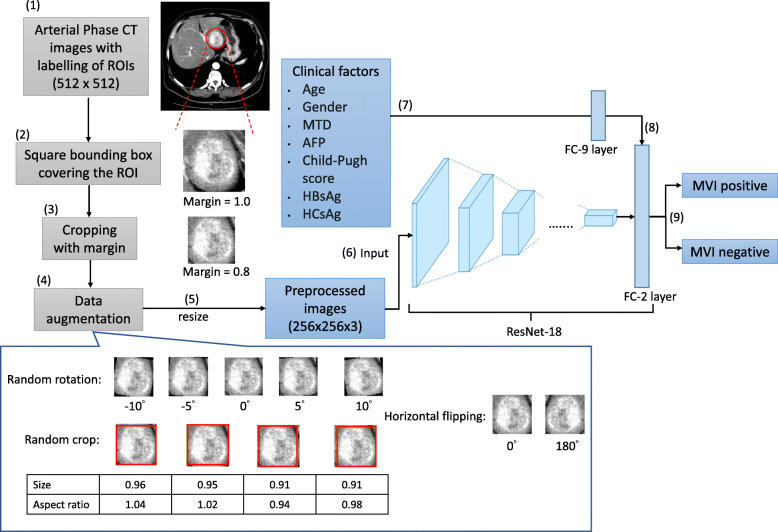
This flow chart summarizes the steps performed in the development of a deep learning-based framework model to preoperatively predicting MVI in HCC. The sequences and details of these steps are: (1) labeling of region of interest (ROI; red circle) on an arterial phase CT image; (2) covering the ROI with a square bounding box; (3) cropping ROI with a margin of 0.8; (4) performing data augmentation, including random rotation, random cropping and horizontal flipping, to increase variation of tumor appearance in the training set; (5) resizing all preprocessed images to 256x256x3; (6) utilizing preprocessed images as inputs for ResNet-18 model training; (7) inputting patients’ clinical factors to an fully connected layer with 9 units (FC-9); (8) concatenating output of the FC-9 layer into the last FC layer (with 2 units) of ResNet-18; (9) predicting MVI positive or negative as the outcome. In step (4), some examples of images in data augmentation were provided. For random rotation (images randomly rotating at − 10 to 10 degrees) and horizontal flipping, the rotating angle is shown below each image. For random cropping (images randomly cropped with sizes of 0.8–1.0 and aspect ratios of 0.95–1.05), the cropped size and aspect ratio is shown below each image. The red square box marks out the resulted image with the corresponding cropped size and aspect ratio. CT = computed tomography; ROI: region of interest; MVI = microvascular invasion; MTD = maximum tumor diameter; AFP = alpha-fetoprotein; HBsAg = Hepatitis B surface antigen; HCsAg = Hepatitis C surface antigen; FC = fully connected

Labeling of regions of interest (ROIs) on all AP slices was performed by a radiologist with 15 years of experiences. A circled ROI, which contained an HCC tumor at the center and was approximately 1 cm larger than the tumor boundary to ensure full coverage of the tumor, was labeled on every CT slice of AP (see Fig. 1). This step was accomplished by the radiologist by carefully comparing all sets of CT images and the pathological findings for each patient. Labeling HCC tumor with a circled ROI on CT slices of AP reduced a substantial amount of labeling time and effort spent by the radiologist compared to delineation of tumor boundary that was performed in other studies [10, 12, 13, 15]. As a consequence, manual tumor contouring, which is usually tedious and time-consuming, was not required in this deep-learning framework. The circled ROI was then converted to a square bounding box fitting the entire tumor and covering the ROI. A square bounding box was used because deep learning models only accept square or rectangular images as inputs.

### Imaging cropping with optimal margin

**Fig. 2:**
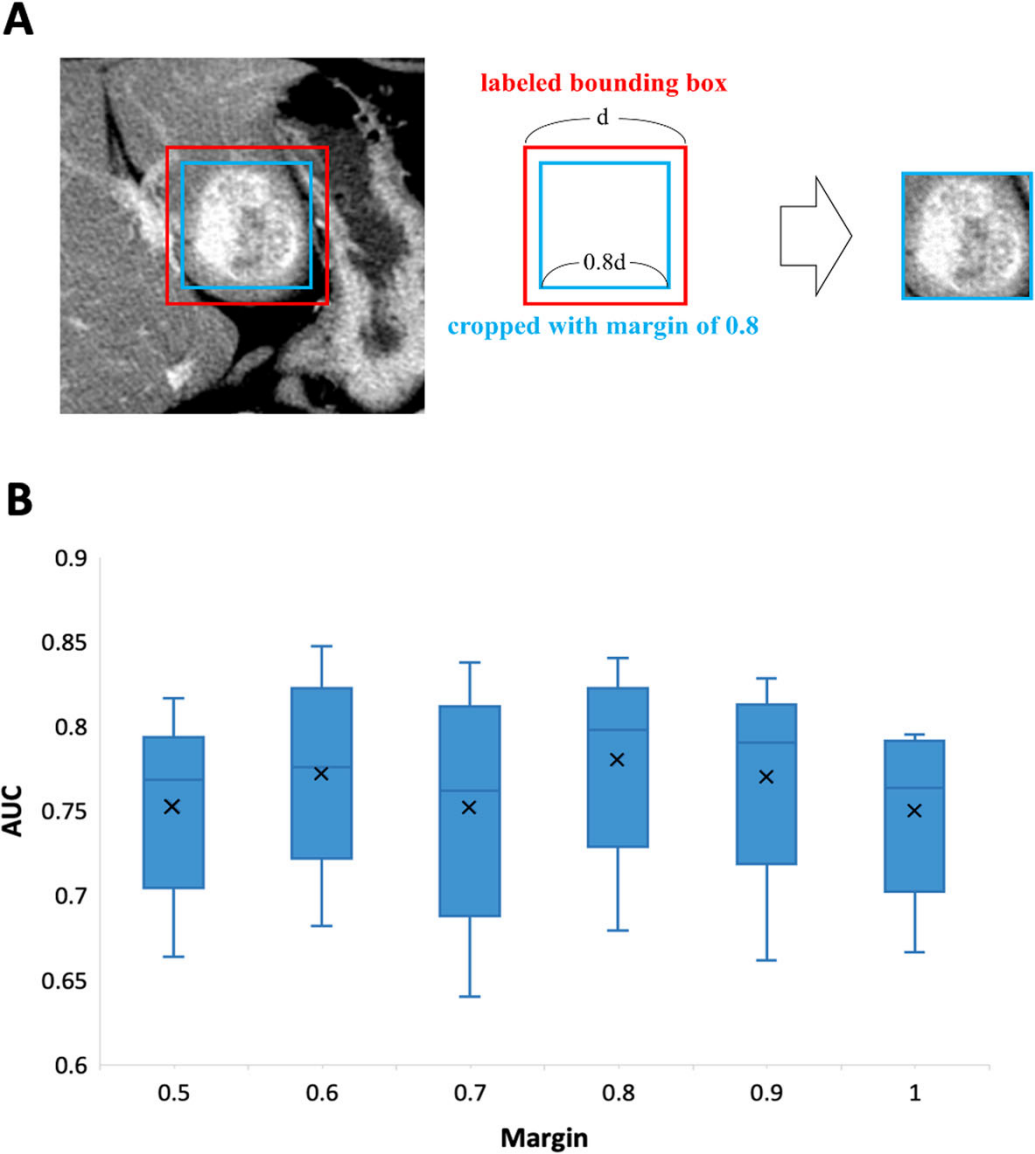
A margin of 0.8 produced the best area under the curve (AUC) in the optimization of cropping margin. (A) An illustration of a cropped image (blue square box) with a margin of 0.8 × the edge length (denoted as d) of the labeled bounding box (red square box). (B) A boxplot showing the performance of ResNet-18 model by using images cropped with marginal values ranging from 0.5 to 1.0. A margin of 0.8 yielded the best mean AUC value from the result of a 5-fold cross validation on the training set. The mean AUC is represented by an ‘x’

The study of Banerjee et al. (2015) revealed that peritumoral regions in CT images provide information, such as hypodense halo and tumor-liver difference, which may be used as indications for the presence of MVI [1]. In contrast, other information, like a large area of air, bone tissues, kidney, great vessels and inferior vena cava, found in these regions can act as artifacts and influence MVI prediction. Therefore, cropping a smaller region from the ROIs could help to remove unnecessary noise or artifacts. Moreover, the margin used to crop the region is critical as a cropped region that is too small may sacrifice some important information relevant to MVI identification. Therefore, a few different values were tested to search for the most optimal margin for cropping. The margin over here is defined as the scale of the edge length of the labeled bounding box. A margin less than 1.0 produces a cropped region that is smaller than the labeled bounding box, as shown in Fig. 2A. We compared the performance of a deep learning model, ResNet-18, with marginal values ranging from 0.6 to 1.0. A margin of 0.8 yielded the best performance in terms of area under the receiver operation characteristic curve (AUC) (Fig. 2B) resulting from a 5-fold cross validation on the training set (*N* = 216). Fig. 2A shows an example of a cropped region with a margin of 0.8. The HCC boundary was found to be fitting nicely within the cropped region (blue square box) implying that the visual information provided in the cropped region is adequate for MVI prediction. All images were processed with a margin of 0.8 and were used in subsequent experiments.

### Data augmentation

After processing all images with the most optimal margin, data augmentation was performed to reduce overfitting of deep learning models. This also increased the data size for model training. Three different types of transformations were applied: (1) randomly rotating at angles varying from -10 to 10 degrees; (2) randomly cropping the images with sizes of 0.8 to 1.0 and aspect ratios of 0.95 to 1.05; (3) horizontally flipping the images with a probability of 0.5. In addition to increasing the data amount, data augmentation also served to increase the variation of tumor visual appearance in size and distortion. This aided in constructing deep learning models which would be robust to the variability of HCC tumors in patients.

### Deep learning model

**Fig. 3:**
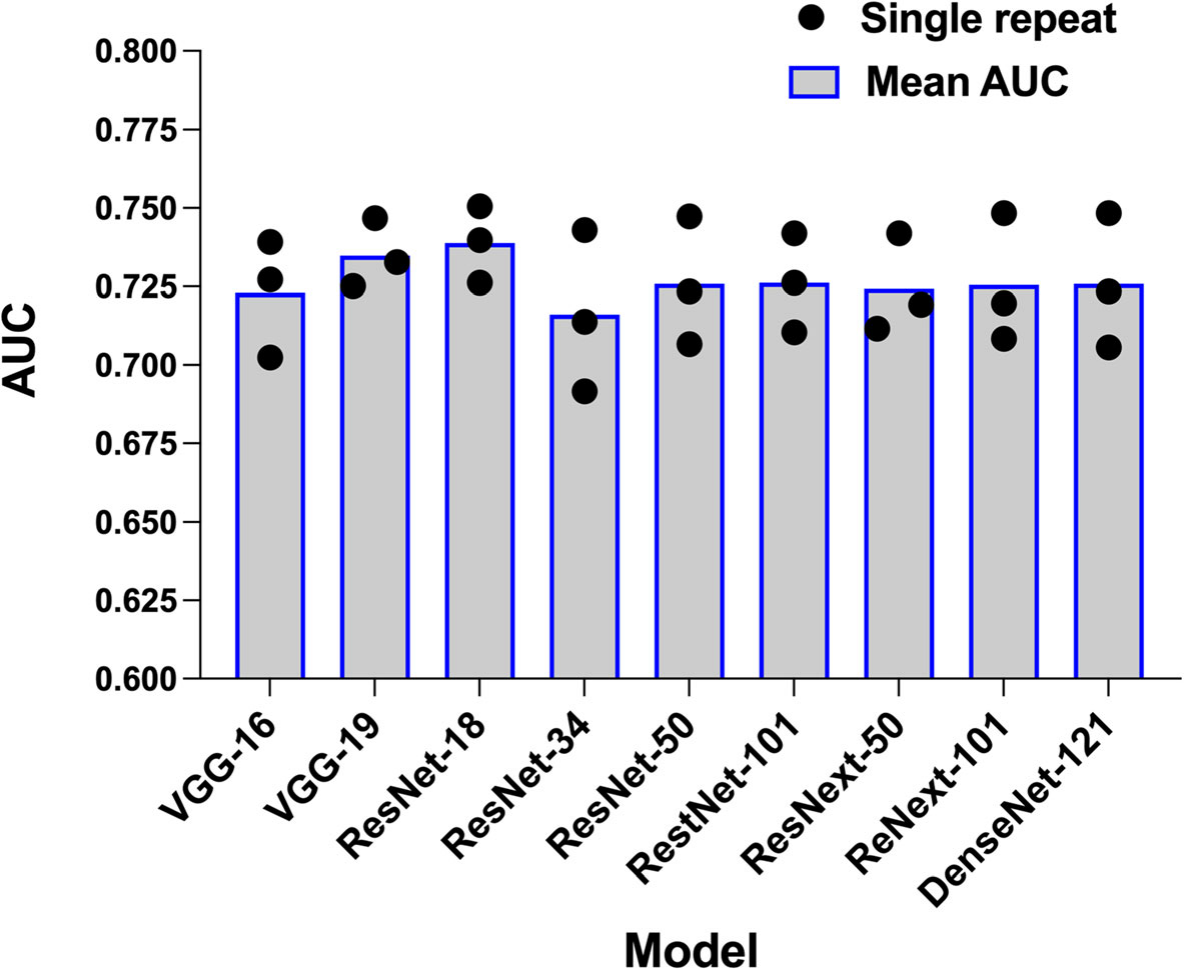
The ResNet-18 model produced the best area under the curve (AUC) compared to other CNN models on the training set. Nine different CNN models (*e.g.* VGG, ResNet, ResNext and DenseNet) and various numbers of layers in these models were tested to search for the best CNN model that utilized CT images of arterial phase to predict MVI in HCC. The numbers of layers are indicated by the postfix numbers in the model names. For each CNN model, three repetitions of 5-fold cross validation were performed on the training set. The dot represents the AUC of each 5-fold cross validation and the bar represents the mean of the three repeats.

Han et al. (2017) [17] demonstrated that a deep learning CNN model, such as GoogLeNet and ResNet, pre-trained on the ImageNet data set is able to benefit visual recognition tasks for medical images. Therefore, we adopted ResNet [18] since it is a CNN model suitable for visual recognition tasks. Moreover, ResNet has been used by other HCC studies to predict surgical response [19] or recurrence [20] with CT images. By taking ResNet-18 as an example, the postfix number in ResNet-18 indicates the number of layers of computational blocks in the neural network. A larger number of layers is usually required for a neural network to handle visual recognition tasks with high complexity. However, a deeper neural network tends to have higher risk of being overfitting. To search for an optimal number of layers and a better CNN model, we tested ResNet models with different numbers of layers as well as other CNN algorithms, *e.g.* VGG [21], ResNeXt [22], and DenseNet [23], using the training set (Fig. 3).

### Machine learning model

In addition to deep learning models, SVM models were developed to be used for comparisons. SVM was applied by Ma et al. (2019) to build a model that utilized radiomics nomograms to preopratively predict MVI in HCC. Moreover, as the output of the last convolutional layer in the trained ResNet-18 model for AP images contained a vector of 512 imaging features, we extracted these imaging features and concatenated them with CFs. Two SVM models were constructed: one was built with CFs and the other one was built with both CFs and imaging features. The SVM model built with CFs was for the purpose of testing if CFs without imaging features would be sufficient to make accurate prediction. On the other hand, the SVM model built with both CFs and imaging features was compared to the RestNet-18 model trained with CFs and AP images. Eventually, the performance of the constructed SVM models was compared to the performance of the developed ResNet-18 models.

### Implementation

#### Deep learning

After applying image cropping and subsequent data augmentation, all AP slices were resized to 256×256. The input image’s size of the pre-trained RestNet-18 model was changed from 224x224 to 256x256. During transfer learning, no layer was frozen and all layers before the final fully connected layer were initialized to the pre-trained model that was trained on the ImageNet data set. Moreover, we used a stochastic gradient descent optimizer on cross-entropy loss with a mini-batch size of 8, a weight decay of 0.00001, a momentum of 0.9, and a learning rate of 0.0003.

#### Machine learning

For SVM model built with CFs, a linear kernel, C value of 0.1, gamma value of 1 and tolerance of 0.001 were used. For SVM model built with CFs and imaging features, a radial basis function kernel, C value of 0.1, gamma value of 0.001, tolerance of 0.1, and maximum iteration of 15 were used

### Prediction and metrics for model evaluation

As one patient had multiple CT slices and MVI statuses of all slices might not be predicted to be the same by the model, we aggregated the prediction results per-patient basis by following the guideline of clinical decision workflow adopted by the radiologist. Based on the guideline, as long as one of the slices shows MVI+, this patient will be considered as having an HCC tumor with MVI+. A patient will only be considered as having an HCC tumor with MVI- if all his/her slices show MVI-. This is because an HCC tumor is three-dimensional and the portion with MVI+ might not be captured in every slice.

For model evaluation, AUC, accuracy, sensitivity and specificity scores were computed for all the models developed in this study on the training, validation and external data sets. Before computation of accuracy, sensitivity and specificity scores, the optimal threshold was first determined using Youden’s J statistic from the receiver operation characteristic (ROC) curve of each model. The J statistic consists of differences between the true positive rate and the false positive rate of the ROC curve. The maximum of the differences was considered as the optimal threshold. The optimal threshold was then used as the cut-off for the predicted probability of MVI to compute the metric scores.

### Model explainability

In addition to prediction, explaining or interpreting how a deep learning model makes predictions has become more important recently to obtain trust from physicians, patients, regulators and other stakeholders involved [24]. Therefore, we explored the critical areas or features on an input image that contributed or had impact to ResNet-18 model prediction using the gradient-weighted class activation map (Grad-CAM) [25]. Grad-CAM visualizes critical areas or features on an image ‘focused’ by a model with the representation of attention heatmaps. We set the intensities of heatmaps as the gradients of model outputs with respect to the activation of the last convolutional layer in the ResNet-18 trained with AP images and CFs. The gradients were computed using backpropagation algorithm.

## Results

### Clinical factors

A total of 309 patients were included in both the training and validation sets. After surgeries, these patients were histopathologically identified and classified into two groups: the MVI+ group (96 patients, 31 %) and the MVI- group (213 patients, 69 %). The clinical factors, demographics and statistical comparison of the training and validation sets are reported in Table 1. There were no significant differences between the MVI+ group and the MVI- group in the training and validation data sets. Moreover, there were no significant differences in age, gender, MTD, AFP, Child-Pugh score, HBsAg, or HCsAg between the training and validation sets. Using both the training and validation sets, a multivariate logistic regression analysis was performed to test the association of these clinical factors with MVI status and the results are shown in Table 3. We used multivariate logistic regression for analysis because we had more than one independent variables. The odd-ratios of MTD, AFP and Child-Pugh score were found to be significant (*p <* 0.05). This indicated that MTD, AFP and Child-Pugh score were independently associated with MVI status.

**Table 3:** Multivariate logistic regression of MVI status based on selected features for clinical factors

Variable	Coefficient	OR [95% CI]	*p*-value
Gender	0.18	1.20 [0.62, 2.31]	0.59
Age	0.01	1.01 [0.98, 1.04]	0.46
MTD	0.32	1.37 [1.22, 1.55]	*<* 0.001*
AFP	7.48 × 10^−5^	1.00 [1.00, 1.00]	0.042*
Child-Pugh score			
A	−2.89	0.06 [0.01, 0.34]	0.002*
B	−3.33	0.04 [0, 0.3]	0.002*
C	−3.3	0.04 [0, 0.62]	0.022*
Hepatitis B	−0.16	0.85 [0.39, 1.85]	0.68
Hepatitis C	0.27	1.31 [0.59, 2.9]	0.5

Abbreviations can be referred to Table 1. *OR* odds-ratio; *CI* confidence interval; * *p <* 0.05 indicates a statistical significance.

A total of 164 patients were included in the external data set. Although patients in the external data were referred from multiple medical centers, all of them received subsequent surgical treatment at CMUH. Based on the histopathological results, 39 patients (24 %) were MVI positive and 125 (76 %) patients were MVI negative.

**Fig. 4:**
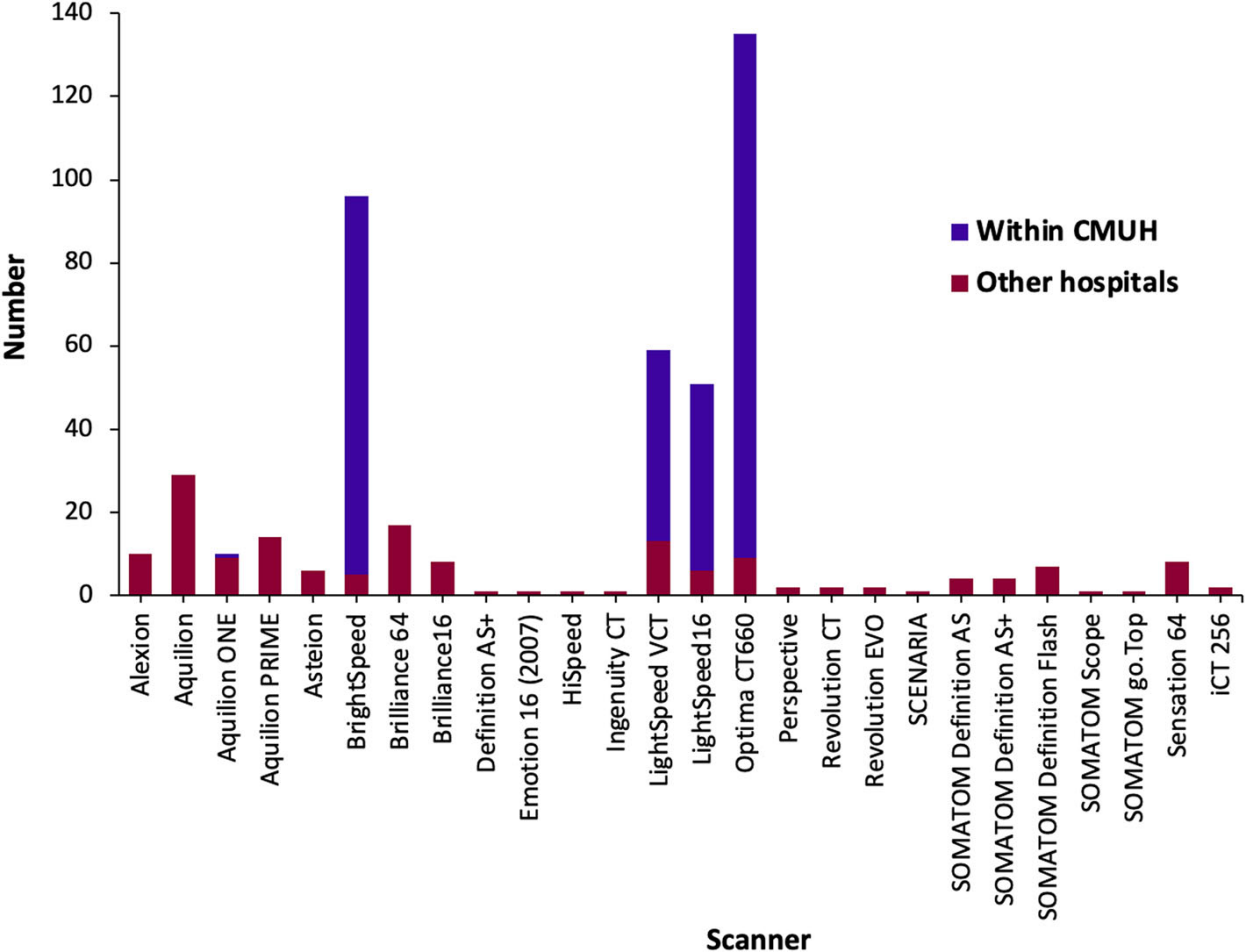
Computed tomography (CT) scanners used by China Medical University Hospital (CMUH; purple bars) and other hospitals (red bars) for CT imaging were manufactured by various different vendors. The bar indicates the number of CT images of arterial phase that were scanned using the particular scanner. CT images within CMUH contained arterial phase images in the training and validation sets while CT images from other hospitals included images in the external data set. There was a larger variety of scanners that were used by other hospitals compared to CMUH for imaging. CT images taken at CMUH were mainly scanned using scanners of these four types: BrightSpeed, LightSpeed VCT, LightSpeed16 and Optima CT 660

The clinical factors and demographics of the external data set are reported in Table 2. The CT images of patients referred from other hospitals were acquired using a larger variety of CT scanners with disparate manufacturing vendors compared to patients within CMUH (Fig. 4). As a consequence, the external set is useful in verifying the robustness of our developed model in making an accurate prediction.

### Construction of CNN (ResNet-18) models for MVI prediction

**Fig. 5:**
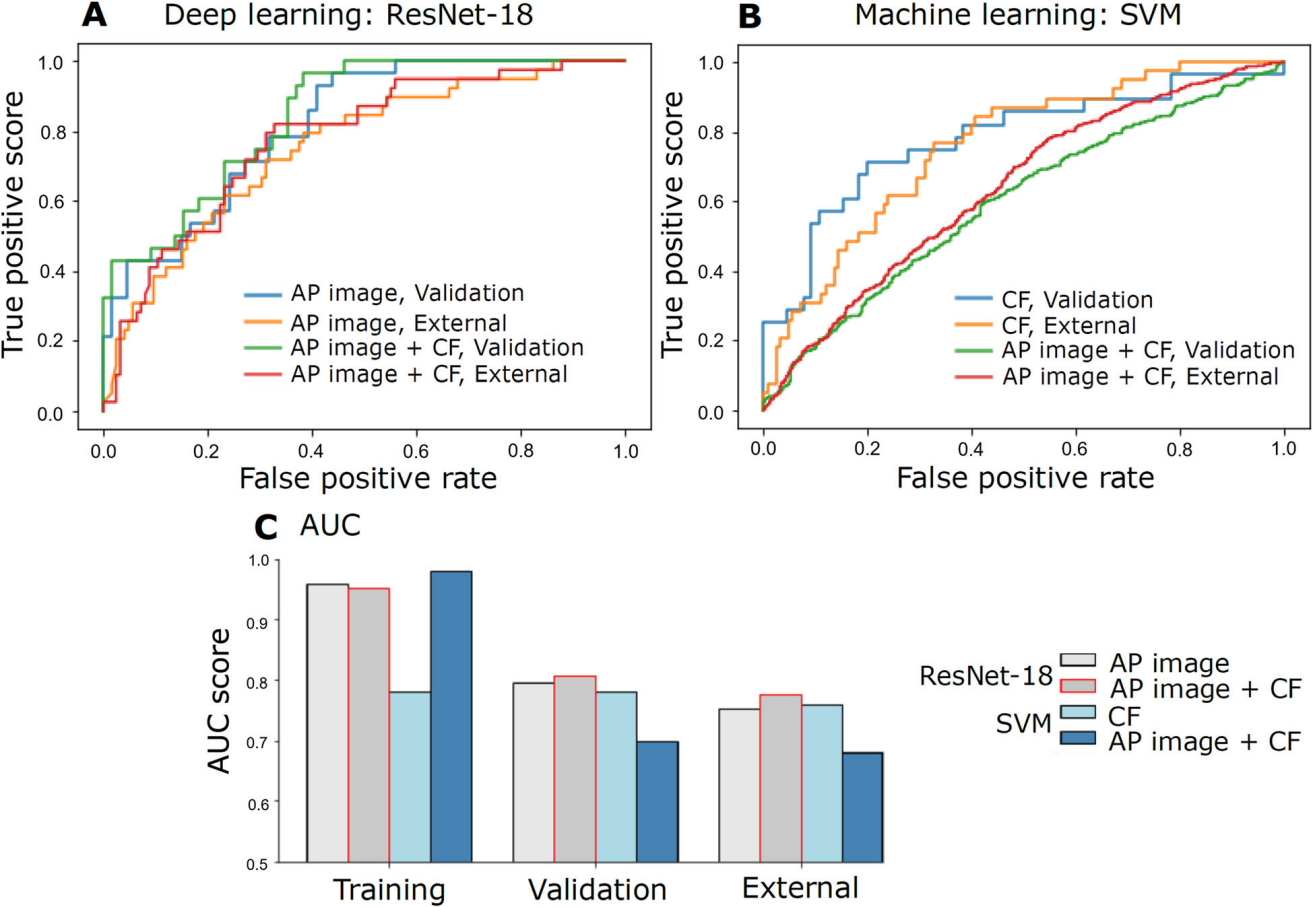
The developed ResNet-18 model utilizing arterial phase (AP) images and clinical factors (CF) produced the highest area under the receiver operation characteristic curve (AUC) on the validation and external data sets. The receiver operation characteristic curves of (A) the 2 deep learning and (B) the 2 machine learning models on validation and external data sets. (C) The AUC scores of the four models on training, validation and external data sets. During ResNet-18 model development, 5 repetitions of model training and validation were performed. The best of the 5 repetitions that produced the highest AUC on the validation set was then used to make predictions on the external data set. No repetition was performed in machine learning model development because their predictions were stable and consistent with repetitions. The receiver operation characteristic curves in (A) were plotted from the models that had the highest AUC among the 5 repetitions. SVM = support vector machine

In this current study, a ResNet-18 model utilizing patients’ AP CT images and CFs was developed to predict MVI pre-operatively. The detailed process of constructing this ResNet model, including image cropping, data augmentation, resizing and image pre-processing, is shown in Fig. 1. Another ResNet-18 model utilizing only AP CT images was also constructed for performance comparison. We ran model training and validation 5 times for both these ResNet-18 models. The ROC curves and mean AUC scores of these ResNet-18 models (AP image vs. AP image + CF) are presented in Fig. 5A & C. The accuracy, sensitivity, specificity and AUC score of these models (the best among 5 repetitions) are reported in Table 4 for training, validation and external sets. The model developed with both the patients’ AP images and CFs produced an AUC score of 0.85 while the model for AP images had an AUC score of 0.82 on the validation set. For the external set, the AUC score of the model for AP images and CFs was 0.78 and the model for CFs was 0.75. The AUC score (0.78) of the external set was close to the mean AUC score (0.81) of the validation set indicating that the ResNet-18 model for AP images and CFs was able to generalize to patients who had their CT imaging obtained in other hospitals. This also implied that the model can be applied to predict MVI status using CT images scanned using scanners manufactured by various different vendors (Fig. 4). When looking at the results of multiple metrics on the external set (Fig. 5), the ResNet-18 model utilizing both AP images and CFs for MVI prediction had an overall higher accuracy, sensitivity and specificity and AUC scores than the one utilizing only AP images for MVI prediction.

**Table 4:** Accuracy, sensitivity, specificity and AUC scores of MVI predictive models for training, validation and external sets

Model	Training set				Validation set				External set			
	Accuracy	Sensitivity	Specificity	AUC	Accuracy	Sensitivity	Specificity	AUC	Accuracy	Sensitivity	Specificity	AUC
ResNet-18(AP)	0.95	0.91	0.97	0.98	0.68	0.96	0.56	0.82	0.66	0.8	0.62	0.75
ResNet-18(AP + CF)	0.97	0.94	0.98	0.97	0.72	0.96	0.62	0.85	0.71	0.82	0.67	0.78
SVM(CF)	0.71	0.82	0.66	0.78	0.77	0.71	0.8	0.78	0.7	0.77	0.67	0.76
SVM(AP + CF)	0.93	0.92	0.93	0.98	0.6	0.93	0.46	0.7	0.57	0.9	0.47	0.68

ResNet-18 model built with AP images and CF generalized well and produced the best metric scores on the external validation set. *SVM* support vector machine; *AP* arterial phase; *CF* clinical factors; *AUC* area under the curve.

### Construction of SVM models for MVI prediction

Next, we trained SVM algorithms to use either CFs or CT imaging features and CFs to pre-operatively predict MVI. A SVM model for CFs was constructed because we were interested to test if CFs only were sufficient to predict MVI. We were also interested to find out if a hybrid model, *i.e.* extracted CT imaging features from a deep learning model were used as inputs for a machine learning model [26, 27], would perform as good as the ResNet-18 model. Therefore, we extracted 512 imaging features from the ResNet-18 model that was developed with AP images only and then fused with their corresponding patients’ CFs. These data were then used as inputs to develop a SVM model for AP images and CFs. The ROC curves and AUC scores of the constructed two SVM models (CF and AP image + CF) are presented in Fig. 5B & C. Meanwhile, the accuracy, sensitivity and specificity of these models are reported in Table 4. Unlike the ResNet-18 models, the ROC curves of these two SVM models were less overlapping (Fig. 5B). The SVM model for CFs only produced higher AUC scores on the validation and external sets than the SVM model for both AP images and CFs (Fig. 5C). Although AUC score (Fig. 5C and Table 4) could achieve above 0.95 on the training set, the SVM model using both AP images and CFs for MVI prediction was unable to generalize well to the validation and external sets (see Fig. 5C, Table 4). This indicated that the SVM model was overfitting. On the other hand, overfitting was less observed in the SVM model using only CFs for MVI prediction.

### Comparison of CNN (ResNet-18) versus SVM models

In the training set, all ResNet-18 and SVM models could achieve AUC scores above 0.95 except the SVM model that used only patients’ CFs for MVI prediction (Fig. 5C). The ROC curves of the SVM model that used both AP images and CFs for MVI prediction (fig 5B) on the validation and external sets revealed that its performance was the worst among these four models. Furthermore, when comparing performance of the four models on the validation and external sets, the AUC scores of the ResNet18 model for AP images and CFs were the highest while the SVM model for AP images and CFs were the lowest. The multiple metric scores, except sensitivity, of the SVM model for AP images and CFs on the validation and external sets (Table 4) were also not as high as the respective scores of the other models. Overall, the ResNet-18 model utilizing both AP images and CFs for MVI prediction had the highest AUC scores (Fig. 5C) and most of its multiple metric scores (Table 4) were also higher than the other models.

### Explainability of ResNet-18 model

**Fig. 6:**
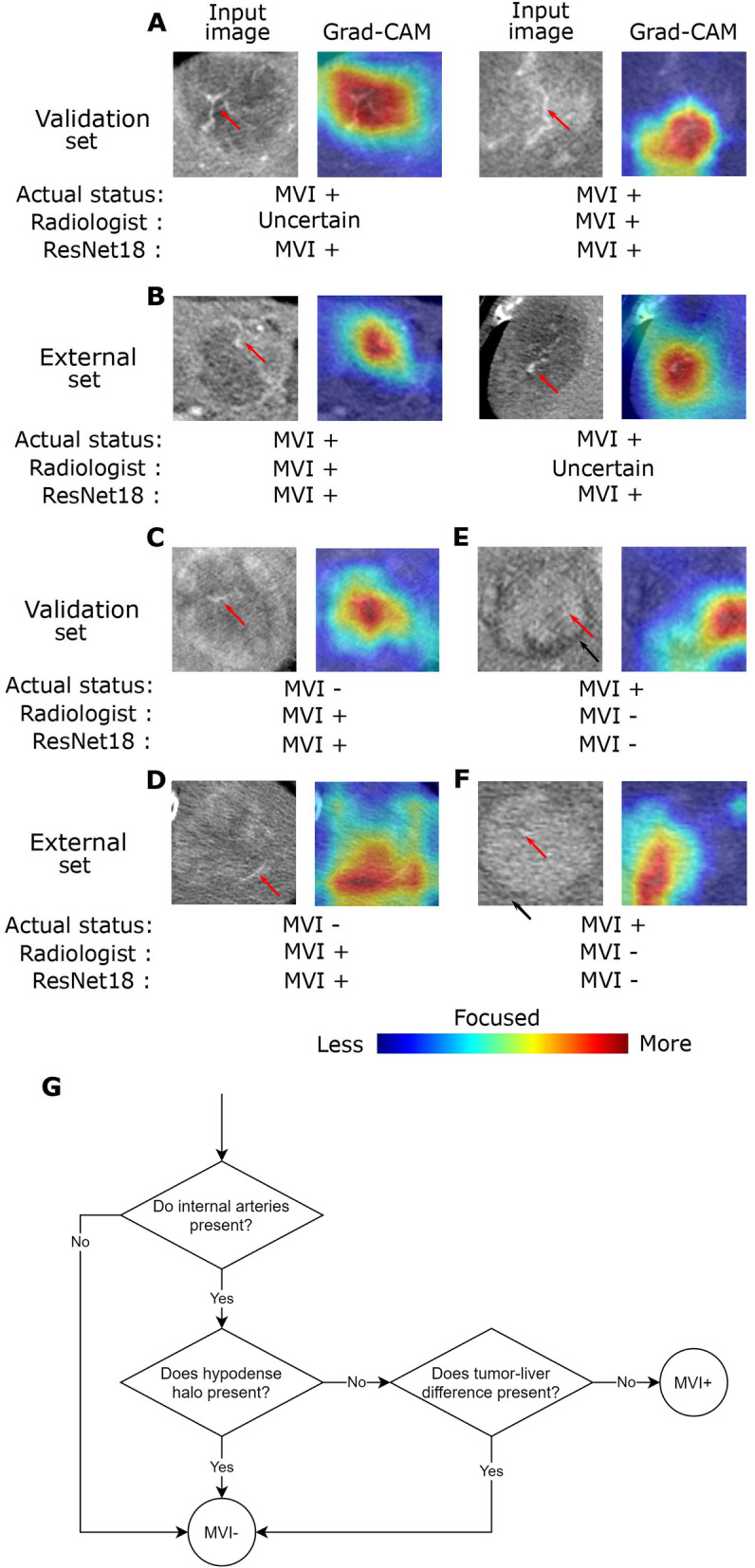
The developed ResNet-18 model for AP image and CF learned MVI relevant features that were similar to the conditions used by radiologists in estimating MVI clinically. Representative examples of attention heatmaps were generated by using the gradient-weighted class activation mapping (Grad-CAM) method for (A-B) true positive, (C-D) false positive, and (E-F) false negative in the validation and external sets. Heatmaps are standard jet colormaps and overlapped on the original input image. Red arrows indicate presence of internal arteries and black arrows indicate presence of hypodense halo. The actual MVI status based on the histopathological results, radiologist’s MVI estimation and MVI prediction of the ResNet-18 model are shown below each representative example. (G) An illustration of the clinical decision workflow applied by radiologists in estimating MVI

Four representative CT images of true positive (Fig. 6A-B) from the validation and external sets, and two false positives (Fig. 6C-D) and false negative (Fig. 6E-F), each from the validation and external sets, as well as their respective attention heatmaps were presented in Fig. 6. We noticed that the ResNet-18 model for AP images and CFs identified MVI similar to the logic reported by Banerjee et al. (2015) in determining RVI. RVI, a contrast-enhanced CT biomarker of MVI derived from a 91-gene HCC “venous invasion” gene expression signature, was demonstrated to have a strong relationship with histological MVI and predict MVI independently [28]. A three-trait decision tree (refer to Fig. 1B in [1]), which involves defining internal arteries, hypodense halo and tumor-liver differences on CT images, was used to describe the strategy used by the radiologist to identify MVI. A representative of this three-trait decision tree was re-drawn and shown in Fig. 6G. According to Banerjee et al. (2015), an HCC tumor on a CT image is identified as MVI positive if its visual appearance satisfies these three conditions sequentially: (1) presence of internal arteries; (2) no hypodense halo; and (3) no tumor-liver difference. As shown in Fig. 6A-D, these representative images in the validation and external sets were classified as MVI positive by our ResNet-18 model (red arrows indicate presence of internal arteries) because they satisfied all the three conditions. On the other hand, the two representative images in Fig. 6E-F satisfied either one or two out of these three conditions and were classified as MVI negative by our RestNet-18 model (black arrows indicate presence of hypodense halo). For Fig. 6A-B, MVI status predicted by the ResNet-18 model were consistent with the real MVI status, which was based on the histopathological results. MVI statuses estimated by the radiologist were uncertain for Fig. 6A (left panel) and B (right panel) because tumor-liver differences were found to be vague. This provided examples showing the advantage of ResNet18 model in aiding to predict MVI accurately. Meanwhile, MVI predictions made by the ResNet-18 model were different from the actual MVI status in Fig. 6C-F. Although conflicts were found between the actual histopathological diagnosis of MVI and the prediction of ResNet-18 model in these cases, MVI estimations made by the radiologist, based on the strategy presented in Fig. 6G, were consistent with the outcomes of our ResNet-18 model. Patients would be recommended to undergo a more detailed checking for further assessment of MVI status when the result of histopathological diagnosis is different from the ResNet-18 model prediction.

## Discussion

A preoperative noninvasive assessment of MVI is helpful in assisting surgeons to determine the tumor resection area as well as guiding subsequent treatment methods [15]. Previous studies used radiological and/or radiomics features extracted from CT images to construct regression or machine learning models to predict MVI status preoperatively in HCC patients [10–13]. Moreover, patients’ clinical variables, *e.g.* age, history of hepatic virus infection, AFP, etc., were also used in the MVI predictive model development in other studies [10, 12, 16, 29]. Radiological features, such as MTD, number of tumors, tumor margin, internal arteries, were obtained from image analysis performed by experienced radiologists who were blinded to the pathological, clinical data and MVI status [12, 15]. Meanwhile, radiomics is defined as a quantitative high-throughput extraction method used for converting medical images into high-dimensional data set for feature extraction [30, 31]. It has gained increasing attention in recent years because of its capability in decoding tissue pathology and unveiling the “hidden” data which may be invisible to radiologists’ or clinicians’ eyes [32, 33]. However, the applications of both radiological and radiomics features are limited by manual and tedious tumor contouring, which increased radiologists’ workload, before feature extraction. Instead of using radiological and/or radiomics features, CT images with simple tumor labeling and without the need of manual tumor contouring were demonstrated in this study (refer to step 1 in Fig. 1). As a consequence, this not only saves time but also reduces bias that could be due to human selection and variable factors in experiences.

### Comparison to other studies

#### Model performance

Table 5 summarizes the MVI predictive performance of machine and deep learning models developed by other studies [10, 15] and this study. The accuracy, sensitivity, specificity and AUC scores of training and validation set, as well as the case number used in the studies are shown in the table. Studies performed by Ma et al. (2019) and Jiang et al. (2021) were selected for comparison because machine and deep learning models for MVI prediction in HCC were constructed. Meanwhile, other studies that developed MVI predictive models using regression [10–12] were not included in this comparison. In the training set, a 3D-CNN model developed by Jiang et al. (2021) had the highest AUC score (0.98), which is similar to the AUC score of our ResNet-18 model for AP images. This 3D-CNN model had the highest accuracy (0.85) and AUC (0.91) scores in the validation set as well. In the validation set, although the AUC score (0.85) of our ResNet-18 model for AP images and CFs were slightly lower, this ResNet-18 model had the highest sensitivity (0.96) among all the compared models. This indicated that our ResNet18 model had the highest proportion of MVI positives that were correctly identified in the validation set. Having high sensitivity in preoperative assessment of MVI is actually more essential and critical than having high specificity (*i.e.* the proportion of MVI negatives that are correctly identified) because correctly identifying MVI-positive patients would help them in receiving more suitable surgical treatment, *e.g.* LT [34], and even increase their subsequent survival years [35, 36]. This would also assist surgeons in making the decision of whether a larger resection area around the lesion should be performed to remove as much cancerous tissues as possible and might thus aid in reducing HCC recurrence in MVI-positive patients.

**Table 5:** Accuracy, sensitivity, specificity and AUC scores of MVI predictive models for training and validation sets

Model	Patient number (Train: Valid)		Training set				Validation set		
		Accuracy	Sensitivity	Specificity	AUC	Accuracy	Sensitivity	Specificity	AUC
SVM with LASSO in Ma et al. (AP)	157 (117:47)	0.72	0.62	0.77	0.70	0.68	0.61	0.72	0.68
SVM with LASSO in Ma et al. (AP + PVP + DP)	157 (117:47)	0.78	0.74	0.81	0.85	0.6	0.41	0.7	0.62
SVM with LASSO in Ma et al. (AP + PVP + DP + CF)	157 (117:47)	0.84	0.76	0.88	0.88	0.66	0.5	0.76	0.68
XGB in Jiang et al. (Radiological + radiomics + CF)	405	–	–	–	0.97	0.85	0.82	0.89	0.9
3D-CNN in Jiang et al. (AP + PVP + DP)	405	–	–	–	0.98	0.85	0.93	0.76	0.91
ResNet-18 in this study (AP)	309 (216:93)	0.95	0.91	0.97	0.98	0.68	0.96	0.56	0.82
ResNet-18 in this study (AP + CF)	309 (216:93)	0.97	0.94	0.98	0.97	0.72	0.96	0.62	0.85

Scores that were not reported in the study were represented by ‘-’. *SVM* support vector machine; *LASSO* least absolute shrinkage and selection operator; *3D-CNN* three-dimensional convolutional neural network; *XGB* extreme gradient boosting; *AP* arterial phase; *PVP* portal venous phase; *DP* delay phase; *CF* clinical factors; *AUC* area under the curve.

#### Phase of CT images

In addition to model performance, the phase of CT images used in this study was also different from the other two studies. CT images of one phase, *i.e.* AP, were used in the development of our ResNet-18 model while CT images of three phases, *i.e.* AP, PVP and DP, were used in the SVM model developed by Ma et al. (2019) and the 3D-CNN model constructed by Jiang et al. (2021). Meanwhile, Ma et al. (2019) had another SVM model which used CT images of AP only for MVI prediction. However, the sensitivity and AUC scores of this model were much lower than our ResNet-18 model that used AP images for MVI prediction. On the other hand, although CT images of DP and PVP were not used, the AUC score (0.845) of our ResNet-18 model for AP images and CFs was more than 80 % in the validation set. Meanwhile, the 3D-CNN model that used CT images of AP, PVP and DP for MVI prediction had an AUC score of 0.906. CT images of PVP and DP may provide additional information relevant to MVI status, this does not, however, aid in improving the predictive performance dramatically. As shown in Table 5, Ma et al. (2019) constructed SVM models using CT images of AP and CT images of 3 phases (AP, PVP and DP). The AUC score of the SVM model for 3 phases was higher than the AUC score of the SVM model for AP images in the training set but the AUC score of the SVM model for 3 phases was lower than the AUC score of the SVM model for AP images in the validation set. This showed that the model developed with 3 phases might have problem in generalization. Moreover, temporal delays set for PVP and DP imaging usually vary from hospital to hospital. This may eventually contribute to data drift in CT images and degrade model performance when deploying the model in other hospitals. Our deep learning-based framework will likely have improved generalizability by using CT images of AP as inputs because the criterion for scanning AP images is consistent and standardized across hospitals. The generalizability and robustness of our ResNet-18 model was verified by its performance on the external set approaching its performance on the validation set.

#### 2D-CNN versus 3D-CNN

As a hepatic tumor has three-dimensional (3D) volume, a small volume sample of multiple CT slices of AP, PVP and DP were used as inputs to construct a CNN model in the study performed by Jiang et al. (2021). When 3D data were used as inputs in a model training, the developed CNN model is a 3D-CNN model. In contrast, one CT slice at a time from the volume was used as the input during ResNet-18 model training in this study. As a CT slice is two-dimensional (2D), our developed ResNet-18 model is a 2D-CNN model. Although hepatic tumors are 3D and it should be reasonable to use 3D volume samples as inputs for 3D-CNN classification tasks, such as malignant tumor identification or MVI detection, a 3D-CNN model is substantially larger than a 2D-CNN model and requires more complicated preprocessing steps [37]. A 3D-CNN model not only has exponentially more parameters, it also requires more training data, training time and storage space [38]. Furthermore, to elevate model’s learning ability, a better and more advanced computing hardware is essentially important to support the whole training process. The ResNet-18 model for AP images and CFs, which is a 2D-CNN model, was compared to a 3D-CNN model in terms of speed and memory usage (Table 6). The running speeds of these two CNN models were tested separately on a NVIDIA GeForce GTX 1650 graphic processing unit (GPU) with 4 GB video RAM, an Intel Core i7-9700 central processing unit (CPU1), or an Intel Core i5-8265 central processing unit (CPU2). According to Fig. 1 in Jiang et al. (2021), 3 volumes of 16x64x64 of CT slices were inputted into their 3D-CNN model. We imitated this by feeding 3D data with similar data size as inputs to a 3D-CNN model. To perform a fair comparison, our ResNet-18 model was given a batch of 16 CT slices as inputs at a time. We also tested our ResNet-18 model with average and maximal slices per patient in our data set as the input sizes. Average or maximal slices per patient serves as a better comparison for a patient-based decision. We repeated the same run on GPU or CPU, respectively, for 100 times to obtain an averaged running speed. For 16 and average slices/patient, ResNet18 model completed the run on GPU and CPU faster than the 3D-CNN model. For maximal slices/patients, ResNet-18 model completed the run on GPU faster than the 3D-CNN model. In addition, ResNet-18 model occupied 50 % less memory footprint than the 3D-CNN model. As a result, a 2D-CNN model has apparently higher efficiency and lower memory requirement than a 3D-CNN model. When the accuracy and AUC scores of a 2D-CNN model attain an acceptable range, it would be more practical and have higher potential than a 3D-CNN model to be deployed as an integrated diagnosis software or applied as an embedded software into an HCC tumor scanning system for preoperative MVI prediction.

**Table 6:** A comparison on running speed and memory footprint for ResNet-18 (2D-CNN) and a 3D-CNN model

	Input size	NVIDIA GTX 1650	Intel i7–9700	Intel i5–8265	Memory usage
3D-CNN in Jiang et al.	16x64x64/patient	0.0154 s	0.5009 s	0.6802 s	90.91 MB
2D-CNN in this study	16 slices/patient	0.0052 s	0.3890 s	0.5069 s	44.69 MB
	6 slices /patient (average)	0.0051 s	0.1546 s	0.2213 s	
	49 slices/patient (max)	0.0082 s	1.1347 s	1.6178 s	

The running speed reported in the table is an averaged speed obtained from 100 runs using NVIDIA GeForce GTX 1650 GPU (with 4 GB dedicated video memory), Intel Core i7-9700 CPU, or Intel Core i5-8265 CPU devices.

### Limitations and future research

In this study, external validation of MVI predictive models was demonstrated by using CT images of AP that were taken at other medical centers. Variation in CT acquisition parameters, *e.g.* the injected dosage and flow rate of Iohexol or Iodixanol and the time of image scanning, as well as a lack of harmonization in the types of CT scanners were noticed when compared across multiple medical centers. Since the same scanning criterion for AP is applied in all centers, AP images obtained at multiple different centers have higher consistency in terms of HU density. Hence, external validation could be reasonably achieved by using AP images as inputs to our MVI predictive models. The problem of data drift, which may degrade models’ predictive performance, could thus be overcome. However, the sample size of the data set used for model development in this study was comparable and not dramatically larger than other studies. Therefore, predictive performance, *e.g.* AUC or accuracy, of our models could be improved by increasing the sample size of training set. In addition to data size, manual labeling of ROIs on CT images was still required and performed by only one radiologist in this study. Hence, it needs to be tested by other radiologists to confirm that the result is reproducible. On the other hand, resizing smaller ROIs extracted from CT images might have introduced noise in the spatial domain. However, using an input size (256x256) larger than the tumor ROIs became necessary because the pre-trained ResNet-18 can only accept images with certain input sizes. The use of 2D slices in this study may also limit the amount of information that could be used by the model for MVI prediction.

## Conclusion

In summary, a deep learning model utilizing CT images of AP and patients’ CFs was built from a pre-trained ResNet-18 model to preoperatively predict MVI in HCC. Powerful MVI predictability was observed even with the use of a simple tumor labeling method as feature extraction was automatically performed by the model. This study also provided evidence showing model external validation on CT images scanned at other hospitals. Last but not least, attention heatmaps obtained from model explainability revealed imaging features relevant to MVI learned by the ResNet-18 model in predicting MVI. These features overlapped with the clinical decision workflow applied by radiologists in estimating MVI.

## Data Availability

The data sets used and/or analyzed during the current study are available from the corresponding author on reasonable request.

## Abbreviations

AFP: Alpha-fetoprotein
AP: Arterial phase
AUC: Area under the receiver operation characteristic curve
CF: Clinical factor
CMUH: China medical university hospital
CNN: Convolutional neural network
CPU: Central processing unit
CT: Computed tomography
DP: Delay phase
FC: Fully connected
Grad-CAM: Gradient-weighted class activation mapping
GPU: Graphic processing unit
HBsAg: Hepatitis B surface antigen
HCsAg: Hepatitis C surface antigen
HCC: Hepatocellular carcinoma
HU: Hounsfield unit
LASSO: Least absolute shrinkage and selection operator
LT: Liver transplantation
MTD: Maximum tumor diameter
MVI: Microvascular invasion
OR: Odds-ratio
PVP: Portal venous phase
Q: Quantile
RFA: Radio-frequency ablation
ROC: Receiver operation characteristic
ROI: Region of interest
RVI: Radiogenomic venous invasion
SVM: Support vector machine
XGB: Extreme gradient boosting
2D: Two-dimensional
3D: Three-dimensional

## References

[1] Banerjee S, Wang DS, Kim HJ, Sirlin CB, Chan MG, Korn RL (2015). A computed tomography radiogenomic biomarker predicts microvascular invasion and clinical outcomes in hepatocellular carcinoma. Hepatology.

[2] Ferlay J, Shin HR, Bray F, Forman D, Mathers C, Parkin DM (2010). Int J Cancer.

[3] Erstad DJ, Tanabe KK. Prognostic and Therapeutic Implications of Microvascular Invasion in Hepatocellular Carcinoma. Ann Surg Oncol. 2019;26(5):1474–93. Available from: https://pubmed.ncbi.nlm.nih.gov/30788629/. 10.1245/s10434-019-07227-9 30788629

[4] Zimmerman MA, Ghobrial RM, Tong MJ, Hiatt JR, Cameron AM, Hong J, et al. Recurrence of hepatocellular carcinoma following liver transplantation: a review of preoperative and postoperative prognostic indicators. Arch Surg. 2008;143(2):182–8; discussion 188. https://pubmed.ncbi.nlm.nih.gov/18283144/. 10.1001/archsurg.2007.39 18283144

[5] Sun WC, Chen IS, Liang HL, Tsai CC, Chen YC, Wang BW, et al. Comparison of repeated surgical resection and radiofrequency ablation for small recurrent hepatocellular carcinoma after primary resection. Oncotarget. 2017;8(61):104571–81. Available from: https://pubmed.ncbi.nlm.nih.gov/29262662/. 10.18632/oncotarget.21604. 10.18632/oncotarget.21604 PMC5732828 29262662

[6] Roayaie S, Blume IN, Thung SN, Guido M, Fiel MI, Hiotis S (2009). A system of classifying microvascular invasion to predict outcome after resection in patients with hepatocellular carcinoma. Gastroenterology..

[7] Cong WM, Bu H, Chen J, Dong H, Zhu YY, Feng LH, et al. Practice guidelines for the pathological diagnosis of primary liver cancer: 2015 update. World J Gastroenterol. 2016;22(42):9279–87. Available from: https://pubmed.ncbi.nlm.nih.gov/27895416/. 10.3748/wjg.v22.i42.9279 PMC5107692 27895416

[8] Feng LH, Dong H, Lau WY, Yu H, Zhu YY, Zhao Y (2017). Novel microvascular invasion-based prognostic nomograms to predict survival outcomes in patients after R0 resection for hepatocellular carcinoma. J Cancer Res Clin Oncol.

[9] Rodr’ıguez-Per’alvarez M, Luong TV, Andreana L, Meyer T, Dhillon AP, Burroughs AK. A systematic review of microvascular invasion in hepatocellular carcinoma: diagnostic and prognostic variability. Ann Surg Oncol. 2013;20(1):325–39. Available from: https://pubmed.ncbi.nlm.nih.gov/23149850/. 10.1245/s10434-012-2513-1 23149850

[10] Ma X, Wei J, Gu D, Zhu Y, Feng B, Liang M (2019). Preoperative radiomics nomogram for microvascular invasion prediction in hepatocellular carcinoma using contrast-enhanced CT. Eur Radiol.

[11] Xu X, Zhang HL, Liu QP, Sun SW, Zhang J, Zhu FP, et al. Radiomic analysis of contrast-enhanced CT predicts microvascular invasion and outcome in hepatocellular carcinoma. J Hepatol. 2019;70(6):1133–44. Available from: https://pubmed.ncbi.nlm.nih.gov/30876945/. 10.1016/j.jhep.2019.02.023 30876945

[12] Peng J, Zhang J, Zhang Q, Xu Y, Zhou J, Liu L (2018). A radiomics nomogram for preoperative prediction of microvascular invasion risk in hepatitis b virus-related hepatocellular carcinoma. Diagn Intervent Radiol..

[13] Ni M, Zhou X, Lv Q, Li Z, Gao Y, Tan Y, et al. Radiomics models for diagnosing microvascular invasion in hepatocellular carcinoma: which model is the best model?. Cancer Imaging. 2019;19(1):60. Available from: https://pubmed.ncbi.nlm.nih.gov/31455432/. 10.1186/s40644-019-0249-x. 10.1186/s40644-019-0249-x PMC6712704 31455432

[14] Nie P, Wang N, Pang J, Yang G, Duan S, Chen J, et al. CT-based radiomics nomogram: a potential tool for differentiating hepatocellular adenoma from hepatocellular carcinoma in the noncirrhotic liver. Acad Radiol. 2021;28(6):799–807. Available from: https://pubmed.ncbi.nlm.nih.gov/32386828/. 10.1016/j.acra.2020.04.027 32386828

[15] Jiang YQ, Cao SE, Cao S, Chen JN, Wang GY, Shi WQ, et al. Preoperative identification of microvascular invasion in hepatocellular carcinoma by XGBoost and deep learning. J Cancer Res Clin Oncol. 2021;147(3):821–833. Available from: https://doi.org/10.1007/s00432-020-03366-9. 10.1007/s00432-020-03366-9 PMC7873117 32852634

[16] Lei Z, Li J, Wu D, Xia Y, Wang Q, Si A (2016). Nomogram for preoperative estimation of microvascular invasion risk in hepatitis B virus-related hepatocellular carcinoma within the milan criteria. JAMA Surg.

[17] Han S, Kang HK, Jeong JY, Park MH, Kim W, Bang WC (2017). A deep learning framework for supporting the classification of breast lesions in ultrasound images. Phys Med Biol.

[18] He K, Zhang X, Ren S, Sun J., Deep Residual Learning for Image Recognition, 2016 IEEE Conference on Computer Vision and Pattern Recognition (CVPR). 2016:770–8. 10.1109/CVPR.2016.90.

[19] Peng J, Kang S, Ning Z, Deng H, Shen J, Xu Y (2020). Residual convolutional neural network for predicting response of transarterial chemoembolization in hepatocellular carcinoma from CT imaging. Eur Radiol.

[20] Wang W, Chen Q, Iwamoto Y, et al. Deep Learning-Based Radiomics Models for Early Recurrence Prediction of Hepatocellular Carcinoma with Multi-phase CT Images and Clinical Data. Annu Int Conf IEEE Eng Med Biol Soc. 2019;2019:4881–4. 10.1109/EMBC.2019.8856356. 10.1109/EMBC.2019.8856356 31946954

[21] Simonyan K, Zisserman A. Very deep convolutional networks for large-scale image recognition. arXiv. 2015;1409.1556v4. Available from: https://arxiv.org/abs/1409.1556v4.

[22] Xie S, Girshick R, Doll’ar P, Tu Z, He K. Aggregated residual transformations for deep neural networks. arXiv. 2016;1611.05431v2. Available from: https://arxiv.org/abs/1611.05431v2.

[23] Huang G, Liu Z, van der Maaten L, Weinberger KQ. Densely Connected Convolutional Networks. arXiv. 2018;1608.06993v5. Available from: https://arxiv.org/abs/1608.06993.

[24] Singh A, Sengupta S, Lakshminarayanan V. Explainable Deep Learning Models in Medical Image Analysis. J Imaging. 2020;6(6):52. 10.3390/jimaging6060052. Available from: https://pubmed.ncbi.nlm.nih.gov/34460598/. 10.3390/jimaging6060052 PMC8321083 34460598

[25] Selvaraju RR, Cogswell M, Das A, Vedantam R, Parikh D, Batra D. Grad-CAM: Visual Explanations from Deep Networks via Gradient-based Localization. Int J Comput Vision. 2016;128(2):336–59. Available from: https://arxiv.org/abs/1610.02391, 10.1007/s11263-019-01228-7.

[26] Han G, Liu X, Zhang H, Zheng G, Soomro NQ, Wang M (2019). Hybrid resampling and multi-feature fusion for automatic recognition of cavity imaging sign in lung CT. Future Generation Comput Syst.

[27] Ozkaya U, Ozturk S, Barstugan M. Coronavirus (COVID-19) classification using deep features fusion and ranking technique. arXiv; 2020. Available from: 10.1007/978-3-030-55258-917*.*

[28] Segal E, Sirlin CB, Ooi C, Adler AS, Gollub J, Chen X (2007). Decoding global gene expression programs in liver cancer by noninvasive imaging. Nat Biotechnol.

[29] Xu Q, Gong Q. Frequency difference beyond behavioral limen reflected by frequency following response of human auditory brainstem. Biomed Eng Online. 2014;13(1):114–27. Available from: http://biomedical-engineering-online.biomedcentral.com/articles/. 10.1186/1475-925X-13-114. 10.1186/1475-925X-13-114 PMC4132204 25108552

[30] Kumar V, Gu Y, Basu S, Berglund A, Eschrich SA, Schabath MB (2012). Radiomics: The process and the challenges. Magn Reson Imaging.

[31] Lambin P, Leijenaar RTH, Deist TM, Peerlings J, De Jong EEC, Van Timmeren J, et al. Radiomics: the bridge between medical imaging and personalized medicine. Nat Rev Clin Oncol. 2017;14(12):749–62. 10.1038/nrclinonc.2017.141. Available from: https://pubmed.ncbi.nlm.nih.gov/28975929/. 10.1038/nrclinonc.2017.141 28975929

[32] Gillies RJ, Kinahan PE, Hricak H (2016). Radiomics: Images are more than pictures, they are data. Radiology.

[33] Aerts HJWL, Rios Velazquez E, Leijenaar RTH, Parmar C, Grossmann P, Carvalho S, et al. Decoding tumour phenotype by noninvasive imaging using a quantitative radiomics approach. Nat Commun. 2014;5:4006. Available from: https://pubmed.ncbi.nlm.nih.gov/24892406/. 10.1038/ncomms5006 PMC4059926 24892406

[34] Mazzaferro V, Llovet JM, Miceli R, Bhoori S, Schiavo M, Mariani L (2009). Predicting survival after liver transplantation in patients with hepatocellular carcinoma beyond the Milan criteria: a retrospective, exploratory analysis. Lancet Oncol.

[35] Shah SA, Cleary SP, Wei AC, Yang I, Taylor BR, Hemming AW (2007). Recurrence after liver resection for hepatocellular carcinoma: Risk factors, treatment, and outcomes. Surgery.

[36] D’Amico F, Schwartz M, Vitale A, Tabrizian P, Roayaie S, Thung S, et al. Predicting recurrence after liver transplantation in patients with hepatocellular carcinoma exceeding the up-to-seven criteria. Liver Transpl. 2009;15(10):1278–87. Available from: https://pubmed.ncbi.nlm.nih.gov/19790142/. 10.1002/lt.21842. 10.1002/lt.21842 19790142

[37] Jiang H, Ma H, Qian W, Gao M, Li Y. An automatic detection system of lung nodule based on multigroup patch-based deep learning network. IEEE J Biomed Health Inform. 2018;22(4):1227–37. Available from: https://pubmed.ncbi.nlm.nih.gov/28715341/. 10.1109/JBHI.2017.2725903 28715341

[38] Xie H, Yang D, Sun N, Chen Z, Zhang Y. Automated pulmonary nodule detection in CT images using deep convolutional neural networks. Pattern Recognit. 2019;85:109–19. Available from: 10.1016/j.patcog.2018.07.031.

